# Effects of caffeine on temporal perception in *Rattus norvegicus*

**DOI:** 10.1371/journal.pone.0304608

**Published:** 2024-05-31

**Authors:** Richard Keen, Dalene Hardy, Belda Jose, H. Neval Erturk

**Affiliations:** 1 Department of Psychology, Converse University, Spartanburg, South Carolina, United States of America; 2 Department of Biology, Chemistry, and Physics, Converse University, Spartanburg, South Carolina, United States of America; University of Nebraska Medical Center College of Medicine, UNITED STATES

## Abstract

We report two studies that tested the effects of caffeine, the world’s most widely used psychoactive drug, on temporal perception. We trained Wistar rats using the Bisection Procedure (Experiment 1) or the Stubbs’ Procedure (Experiment 2) to discriminate between short and long light stimuli. Once training finished, we administered caffeine orally (0, 9.6, and 96.0 mg/kg for Experiment 1 and 0, 9.6, 19.2, and 38.4 mg/kg for Experiment 2) 15 minutes prior to testing. Relative to the control condition, the 9.6 mg/kg condition (Experiments 1 and 2) and the 19.2 mg/kg condition (Experiment 2) resulted in an increase in proportion of choosing the long response. Meanwhile, overall accuracy was not affected by any condition in both experiments. Taken together, these results are consistent with the notion that caffeine, at some doses, speeds up temporal perception. However, it is not clear why the effect disappears at higher doses.

## Introduction

Temporal perception is the awareness of the passage of time following a stimulus event. It is usually inferred by changes in behavior as a function of time. While healthy individuals are expected to display accurate and unbiased temporal perceptions, those with various psychotic disorders, brain pathology or those under the influence of pharmacological agents may display altered temporal perception [1], which is an important component of many psychological and neurological processes, including the perception of music [2], speech [3], and movement [4]. Disruptions in temporal perception can occur in two different, but not entirely independent ways. First is a decrease in the accuracy of perceiving time, possibly due to a factor such as a lack of attention [5]. This is primarily determined by an increase in variability of responding. For example, Brown [5] reviewed 77 timing studies which used divided attention tasks and found that 91% showed more variability in time estimates compared to controls. Second, the disruption in temporal perception may be biased, possibly by either speeding up or slowing down your “internal clock.” For example, methamphetamine and haloperidol have been shown to speed up and slow down temporal perception, respectively using a peak-interval (PI) procedure [6]. In a PI procedure, after a subject is well trained on a fixed-interval (FI) procedure, probe trials are presented in which no food is available while the timed stimulus remains on. Typically, a subject’s peak response rate is close to when the subject was previously reinforced on FI trials. When Buhusi and Meck administered methamphetamine or haloperidol to rats, the rats’ peak response time was significantly earlier or later, respectively, relative to the control condition.

Research shows the neurotransmitter dopamine, which plays a role in various psychological processes, including pleasure, learning, memory, cognition, and motivation, is closely associated with time perception. Dopamine receptors are prominent in the central nervous system (CNS). Although activated by a number of effectors (receptor-receptor interactions or receptor-protein interactions), dopamine is the primary ligand for dopamine receptor activation [7, 8].

According to Meck’s dopamine hypothesis (1983 & 1996), dopamine levels have a direct impact on the internal clock. Increase in dopamine levels (e.g. methamphatimine exposure) in the synapsis cause speeding up of the clock and reduced dopamine levels (e.g. exposure to haloperidol) at the synapsis cause slowing down of the clock [6]. Dopamine receptors form heterodimers with adenosine receptors in the cell membrane. This interaction affects the efficiency of dopamine binding to the dopamine receptors [9, 10]. An adenosine antagonist caffeine (C_8_H_10_N_4_O_2_) is a naturally occurring plant xanthine alkaloid (1,3,7-trimethylxanthine) found in over 60 plants including coffee and cacao beans, tea leaves, guarana berries, and kola nuts. It is the world’s most commonly consumed psychosomatic substance. It is also available as over-the-counter pills as a stimulant. Even though various factors, such as gastric emptying time or caffeine being mixed with sugar or fiber, affect the absorption rate, once consumed caffeine is rapidly and fully absorbed into the bloodstream, therefore the effects of caffeine are experienced shortly after its consumption [11].

The chemical structure of caffeine is similar to adenosine [12]. Adenosine is an endogenous purine nucleotide that functions as a neuromodulator which regulates a diverse range of physiological processes in the cardiovascular, central nervous, and immune systems. The diverse function of adenosine is through the receptors with which it interacts [13–17]. Even though other mechanisms have been proposed [13], adenosine exerts its effect primarily via the activation of one of the four G-protein coupled cell surface receptors called adenosine receptors. Adenosine receptors are comprised of four subtypes, A1, A2a, A2b, and A3. They are found throughout the body in most organs. Research reports that the A2A receptor is mainly expressed in the areas of the brain that are rich in dopamine receptors, such as the striatum globus pallidus, nucleus accumbens, olfactory tubercle, bulbus olfactorius, nucleus nervi acustici, and prefrontal cortex, specifically in the striatonigral-striatoentopeduncular GABAergic pathways. The striopallidal neurons are efferent GABAergic neurons that primarily contains D2 dopaminergic receptors whereas the second subtype, the strionigro-strioentopeduncular neurons, primarily contain D1 dopaminergic receptors [18]. A1 receptors are expressed in both subtypes of neurons, but A2A receptors are expressed only in the striopallidal neurons. This shows that A2A receptors only expressed in the neurons that also express D2 receptors whereas, whereas A1 receptors expressed in the nerve cells that express both D1 and D2 receptors [19–21]. Research shows that the activation of A2A receptors causes a decrease in activation of D2 receptors [22] whereas the A2A receptor inhibition causes an increase in activation of D2 receptors [22, 23]. The effect of dopamine receptors activation on time perception is well established [24–26]. Since (1) previous research has shown that an increase in dopaminergic activity leads to a speeding up of temporal perception and (2) adenosine antagonists (e.g., caffeine) results in the inhibition of the inhibiting effects of adenosine on dopamine, we predict that caffeine consumption will cause disruption to time perception by speeding up of temporal perception. The purpose of this study is to investigate the effects of caffeine on timing using two established timing procedures and to propose a hypothetical model for the mechanism that cause it to alter temporal perception.

## Materials and methods

### Subjects

#### Experiment 1

Fourteen naïve, Wistar rats were used in this experiment, seven male and seven female. They were approximately 60 days old at the commencement of the experiment. All 14 rats were housed in wire cages, and received water ad lib. Rats were fed 15 g of food in their home cage immediately following completion of their daily procedure. Illumination in the colony room was maintained on a 14:10 light: dark cycle.

#### Experiment 2

Nine naïve, female Wistar rats, approximately 60 days old at the commencement of the experiment, were used and housing details were the same as described in Experiment 1. These studies were approved by the Institutional Animal Care and Use Committee of Converse University.

### Apparatus

#### Experiments 1 and 2

Two operant chambers manufactured by Med Associates Inc. were used. All experimental inputs and outputs were controlled and recorded by MED PC-IV software loaded on a PC. Each chamber was enclosed by a sound attenuating box which contained a fan to help circulate air and mask extraneous noises. The chambers’ dimensions were as follows: 30.5 cm x 30.5 cm x 33.0 cm. One wall of each chamber had two retractable levers. A food magazine was located between each lever equidistantly. The food magazine allowed access to 45 mg food pellets (AIN-76A) manufactured by TestDiet®. A diffuse light (28V, bulb #1820) was located 3.75 cm above each lever. General illumination in the operant chamber was provided by a houselight (28V, bulb #1820) located 2 cm from the ceiling on the wall opposite the levers.

### Procedure

#### Experiment 1

Before the experiment began, rats were magazine trained to find food in the operant chamber and then autoshaped to press the levers. Autoshaping sessions lasted for 60 minutes and consisted of 100–116 trials. A trial consisted of the random presentation of a lever (left or right), the presentation of a food pellet, and a 30 second inter-trial interval. The lever presentation lasted for six seconds if not pressed, followed by the immediate presentation of the food pellet. If the rat pressed the lever, the lever immediately withdrew and a food pellet was given.

In the first phase of the experiment rats were trained to distinguish between two and eight second light signals. To accomplish this, the lights above each lever were illuminated for two or eight seconds. After the lights were extinguished both levers were presented. For half of the rats, pressing the left lever after a two second signal resulted in a food pellet reinforcement, as did pressing the right lever after an eight second signal. The reverse was true for the remaining rats. Each trial (i.e., a signal and subsequent response) was followed by a 30 second inter-trial interval. Sessions consisted of 60 trials, 30 short (i.e., 2 s) and 30 long (i.e., 8 s) trials presented in a random order. Rats remained in Phase 1 until they were correct for 80% of the trials for three consecutive sessions or a maximum of 40 sessions.

Phase 2 was the same as Phase 1 with the exception that the reinforcement rate was cut in half. Thus, whenever the rats made the correct choice, they randomly received a food pellet 50% of the time. This phase was included to acclimatize the rats to partial reinforcement that would occur during subsequent phases and lasted 10 sessions.

In Phase 3 of the experiment the Bisection Procedure [27] was used to obtain a baseline psychometric function and lasted for three sessions. In addition to the original two and eight seconds signals, five additional signal durations of equal logarithmic division (2.8, 3.2, 4, 4.9, and 6.3 s) were added. Sessions consisted of 60 total trials. There were 15 trials each of two and eight second signals and the five intermediate durations were presented six trials each. Reinforcement was available for correct choices on two and eight second trials but reinforcement was not available on the intermediate trials.

The fourth and final phase consisted of three experimental conditions: 9.6 mg/kg, 96.0 mg/kg, and the water sham control. Using a within subjects design, every rat participated in each condition in a counterbalanced order. The treatment groups were given either 9.6 or 96.0 mg/kg caffeine dissolved in water. The sham control group received an equivalent amount of distilled water. All solutions were orally delivered in 100 μL quantity 15 minutes before testing. Each condition lasted for three consecutive sessions. There was a recessionary period of two days between each experimental condition. Thus, Phase 4 lasted for a total of 13 sessions: three conditions of three days each and two recessionary periods of two days each. Except for the addition of the caffeine conditions and the number of sessions for each condition, the procedure was the same as described in Phase 3. For all phases, sessions terminated after 60 minutes even if the rat had not completed all of the trials (see Table 1 for a summary of the procedure).

**Table 1:** Procedural summary for Experiment 1.

Experiment 1				
	Trials	Sessions	Caffeine	Reinforcement
Phase 1	2s: 30 trials 8s: 30 trials	80% accuracy for 3 days or 40 sessions	None	100% for correct choices
Phase 2	2s: 30 trials 8s: 30 trials	10 Sessions	None	50% for correct choices
Phase 3	15 trials of 2 & 8s 6 trials of 2.8, 3.2, 4.0, 4.9, & 6.3s	3 Sessions	None	100% for correct choices on 2 and 8s trials
Phase 4	15 trials of 2 & 8s 6 trials of 2.8, 3.2, 4.0, 4.9, & 6.3s	3 sessions for each condition with 2 recessionary sessions in between (13 total sessions)	0.0, 9.6, & 96.0 mg/kg	100% for correct choices on 2 and 8s trials

Experiment 1 used the Bisection Procedure.

#### Experiment 2

Autoshaping and Phase 1 training (i.e., 2 vs 8 s discrimination training) were as described in Experiment 1 except that Phase 1 training consisted of 80 trials per session, 40 short and 40 long. Phase 2 utilized the Stubbs Procedure [28] in which 10 logarithmically spaced intervals (i.e., 2, 2.3, 2.7, 3.2, 3.7, 4.3, 5.0, 5.9, 6.9, & 8 s) were presented eight times per session for a total of 80 trials per session and lasted for 16 sessions. Each rat was assigned one lever as its “short” lever and was reinforced for pressing that lever if the signal duration was less than four seconds and the opposite lever was reinforced for pressing that lever if the signal duration was greater than four seconds. This phase lasted for 16 sessions. Unlike in Experiment 1, correct choices for intermediate durations were reinforced.

In Phase 3, rats were given an oral dose of either 9.6 mg/kg, 19.2 mg/kg or 38.4 mg/kg of caffeine dissolved in water as described in Experiment 1 or they were given a sham water treatment. Each rat received all four conditions in a counterbalanced order. Each treatment lasted for three consecutive days followed by two days off until all four conditions were completed. Thus, Phase 4 lasted for a total of 18 sessions: four conditions of three days each and three recessionary periods of two days each. Except for the addition of the caffeine conditions and the number of sessions for each condition, the procedure was the same as described in Phase 2. For all phases, sessions terminated after 60 minutes even if the rat had not completed all of the trials (see Table 2 for a summary of the procedure).

**Table 2:** Procedural summary for Experiment 2.

Experiment 2				
Phase 1	2s: 40 trials 8s: 40 trials	80% accuracy for 3 consecutive days or 40 sessions max	None	100% for correct choices
Phase 2	8 trials each: 2.0, 2.3, 2.7, 3.2, 3.7, 4.3, 5.0, 5.9, 6.9, & 8.0 s	16 Sessions	None	100% for correct choices
Phase 3	8 trials each: 2.0, 2.3, 2.7, 3.2, 3.7, 4.3, 5.0, 5.9, 8.9, & 8.0 s	3 sessions for each condition with 2 recessionary sessions in between (18 total sessions)	0.0, 9.6, 19.2, & 38.4 mg/kg	100% for correct choices

Experiment 2 used the Stubbs Procedure.

## Data analysis

To determine if caffeine speeds up or slows down temporal perception, the overall probability of choosing the long lever was analyzed with a Repeated-Measures, One-Way ANOVA with planned comparisons (LSD method) of the Control Condition with each Caffeine Condition. A bias to choose the long lever more than the control condition is consistent with a speeding up of temporal perception while a bias to choose the long lever less is consistent with a slowing down. To determine if caffeine disrupts accuracy of temporal perception, the same analysis was conducted on the overall accuracy on trials that were potentially reinforced (i.e., 2 and 8 s trials in Experiment 1 and all trials in Experiment 2).

All statistical tests were conducted using IBM SPSS (v28) and were two-tailed using an alpha level of .05 with no familywise error correction. In Experiment 1, proportion choose long was out of 180 trials per rat while proportion correct was out of 90 trials (2s and 8s trials). In Experiment 2, both proportions choose long and proportion correct were out of 240 trials per rat.

## Results

In Experiment 1, over 99% of the trials were completed in Phases 1 to 3 and 100% were completed in Phase 4. In Experiment 2, over 99% of the trials were completed in Phases 1and 2 while 100% were completed during Phase 3.

### Experiment 1

**Fig 1:**
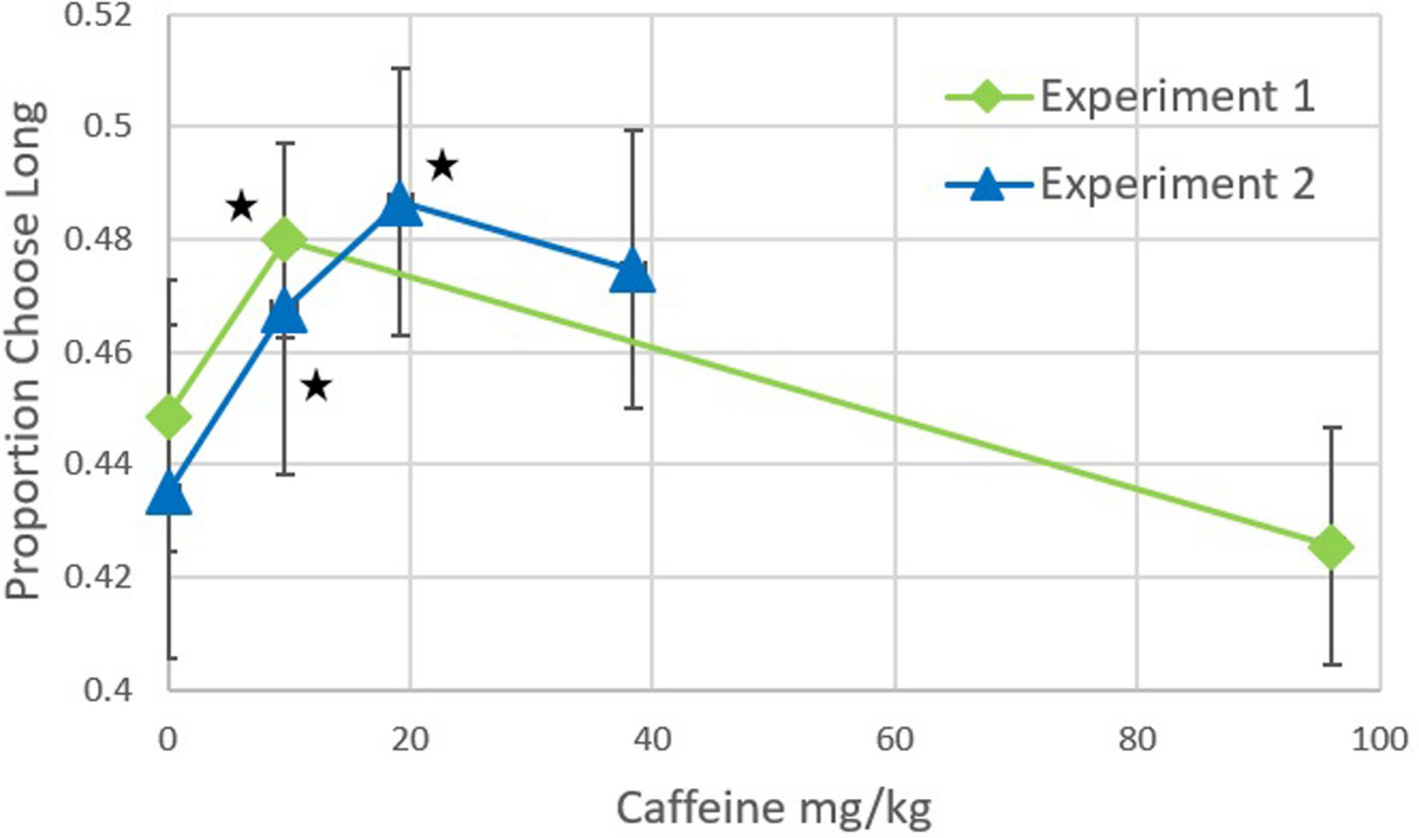
The proportion of choosing long is plotted as a function of how much caffeine was given across Experiments 1 and 2. Shifts up, relative to control, indicate a speeding up of temporal perception while shifts down indicate a slowing down of temporal perception. Error bars represent the standard error of the mean. Stars represent significance at the .05 level for comparisons between each caffeine condition with their respective control condition.

**Fig 2:**
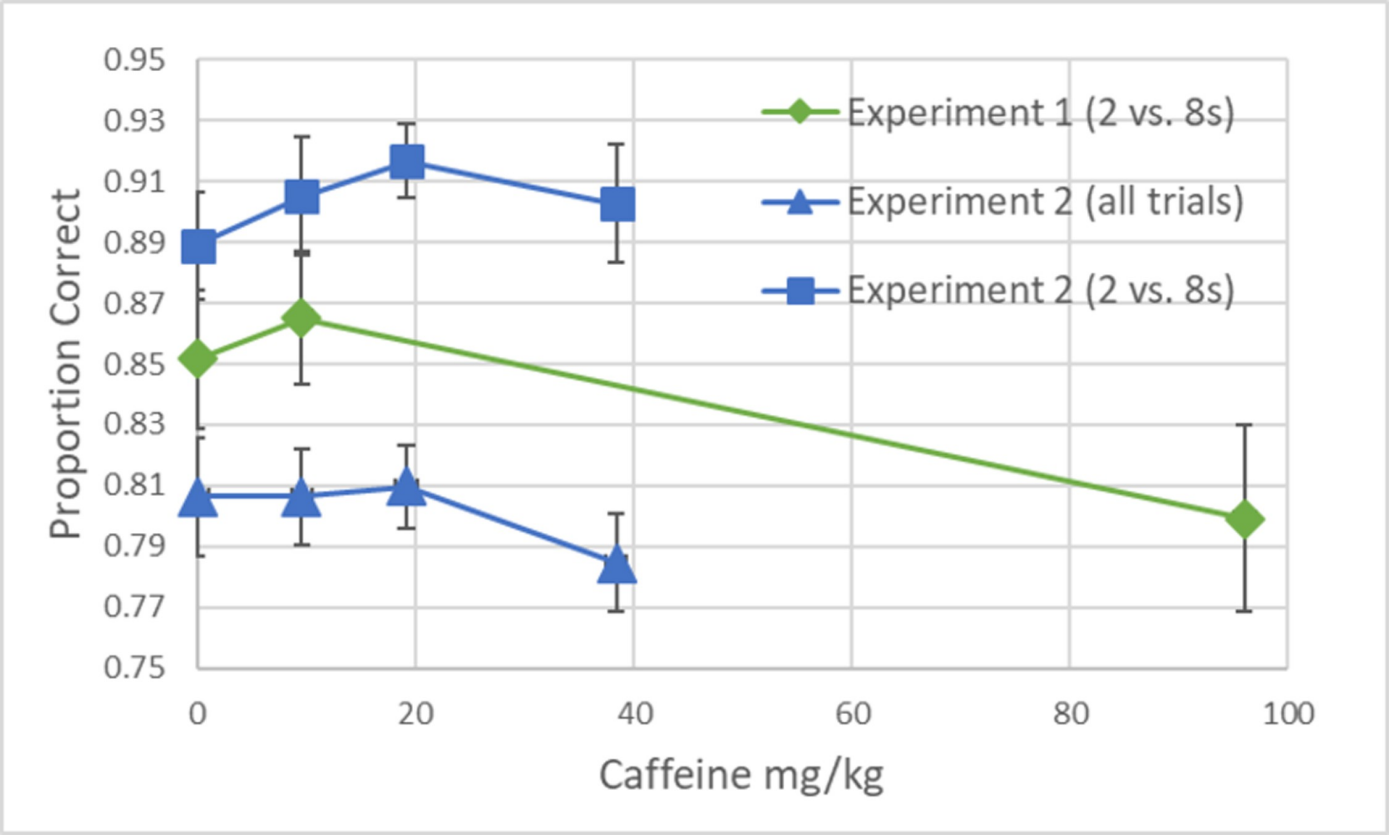
The proportion of correct responses is plotted as a function of how much caffeine is given in Experiments 1 and 2. Correct responses were calculated from trials that were potentially reinforced (i.e., 2 and 8 s trials in Experiment 1 and all trials in Experiment 2). In order to make direct comparisons across experiments, the proportion correct for only the 2 and 8 s trials was also included from Experiment 2. Error bars represent the standard error of the mean.

The ANOVA indicated an overall significant effect for the bias analysis, F(2,26) = 4.88, p = .016, ηp2 = .27. Planned comparisons indicated that the Control Condition (M = .45, SD = .09) was significantly different than the 9.6 mg/kg Condition (M = .48, SD = .07; p = .015) while the Control Condition was not significantly different than the 96.0mg/kg Condition (M = .43, SD = .08; p = .387; see Fig 1). For the Accuracy analysis, F(2,26) = 3.68, p = .016, ηp2 = .22. Planned comparisons indicated that the Control Condition (M = .85, SD = .09) was not significantly different than the 9.6 mg/kg Condition (M = .87, SD = .08; p = .463) nor the 96.0mg/kg Condition (M = .80, SD = .12; p = .387; see Fig 2).

### Experiment 2

The ANOVA failed to find an overall significant effect for the Bias analysis, *F*(3,24) = 2.51, *p* = .083, η_p_^2^ = .24. Planned comparisons indicated that the Control Condition (*M* = .44, *SD* = .09) was significantly different than the 9.6 mg/kg Condition (*M* = .47, *SD* = .09; *p* = .047) and the 19.2 mg/kg Condition (*M* = .49, *SD* = .07; *p* = .028), but not the 38.4 mg/kg Condition (*M* = .48, *SD* = .07; *p* = .150). For the Correct analysis, *F*(3,24) = 0.94, *p* = .439, η_p_^2^ = .11. Planned comparisons indicated that the Control Condition (*M* = .81, *SD* = .05) was not significantly different than the 9.6 mg/kg Condition (*M* = .81, *SD* = .05; *p* = 1.00), the 19.2 mg/kg Condition (*M* = .81, *SD* = .04; *p* = .862), nor the 96.0mg/kg Condition (*M* = .78, *SD* = .05; *p* = .235).

## Discussion

Results from two commonly used timing procedures, Bisection and Stubbs, indicate that orally administered caffeine sped up temporal perception with low to moderate doses (i.e., 9.6 and 19.2 mg/kg), but that the effect disappeared with higher doses (38.4 and 96 mg/kg). Meanwhile, overall accuracy was not significantly affected by caffeine at any of the doses used. These results are consistent and extend the findings by Sankar, Shukla, and Bapi [29] in which they found caffeine sped up temporal perception using a Bisection Timing Procedure.

Individuals whose time perception is sped up may attempt to perform critical actions too early. A particular concern will present itself if a person has a change in the amount of caffeine consumed between different phases of a particular task. For example, an athlete training for competition or a musician practicing for a performance may consistently train without caffeine. If during the competition/performance the individual changes the amount of caffeine consumed in order to maximize the ergogenic effect, then their time perception will be sped up relative to their training periods, resulting in a disrupted performance.

In order to apply the doses used in the current study using rats, interspecies scaling from rats to humans is needed. A simple and fact-based technique for interspecies scaling of drugs is the allometric approach, which is applicable for compounds that are renally excreted, such as caffeine [30]. Rats are one of the most important animal models in biomedical sciences [31], exercise physiology, and sport science [32] due to their biochemical, anatomical, physiological, and genetics similarities to humans [33]. Rats were chosen because (1) there is not a significant first pass effect of oral caffeine in rats and in human, (2) systemic clearance is equal to the metabolic clearance of caffeine both in rats and in humans, (3) gastrointestinal absorption of caffeine is rapid and complete in both species, and (4) an interspecies comparison of dose relationship of caffeine between human and rats are published. Based on this report, rats metabolize caffeine 2–3 times faster than humans. Several studies reported that caffeine and its metabolites have limited ability to pass the blood brain barrier in rats [34]. Previous data showed that humans have a 10 fold difference whereas rats have a 46 fold difference between the plasma and brain caffeine metabolite levels. This difference is particularly important in this study because caffeine exerts its effect via binding to the adenosine receptors located in the brain. Thus, when the doses used in this study are extrapolated from rats to human a 9–14 fold difference should be factored in. The two doses that resulted in a significant speeding up of temporal perception in this study were 9.6 and 19.2 mg/kg body weight. When these doses are extrapolated to humans our 9.6 mg/kg dose corresponds to 0.69 to 1.07 mg/kg and the 19.2 mg/kg dose corresponds to 1.38 to 2.14 mg/kg body weight. Thus, for a 70kg person, the 9.6 and 19.2 mg/kg doses correspond to 48.3 to 74.9 mg and 96.6 to 149.8 mg, respectively. To put these doses into perspective, a 12 oz. can of soda has 34 mg of caffeine and an 8 oz. cup of coffee has 95 mg of caffeine. The two doses that resulted in significant changes in temporal perception are slightly below the 3–6 mg/kg dose that reportedly results in beneficial ergogenic effects [35].

Previous research shows that impairment in duration discrimination affects Reaction Time (RT), which is the time elapsed between the onset of a stimulus and response given to the stimulus [36]. RT can roughly be divided into two processes. The first one, the perception, is being aware of a stimulus. It is the time between the sensory input being received to the brain’s acknowledgment of the perception (e.g., acknowledgement of the sound of an electric horn at the beginning of a swim competition). The second is the processing of perception input by the central nervous system. This is the time between being aware of the stimulus and a motor response being executed in response to the sensory stimulus (e.g., a swimmer moving to commence a race). Since caffeine has been shown to improve reaction time, athletes may take caffeine to improve their performance without considering caffeine’s effect on temporal perception. In some sports (e.g., swimming, sprinting, etc.), the benefit to reaction time may outweigh the disruption to temporal perception, however, in other sports (combat sports, baseball, etc.), the reverse may be true.

Caffeine exerts its ergogenic effect through its antagonistic interaction with two adenosine subtype receptors (A1 and A2A) located on the skeletal muscle membranes [37]. Adenosine subtype receptors are found in all cells of the body. In the central nervous system, A1 receptors are located primarily in the cortex whereas A2A receptors are located in the striatum. In the central nervous system, both A1 and A2A receptors play a role in sleep regulation. Under normal conditions adenosine binds to A1 and A2A receptors to cause sleepiness. A1 receptors are primarily present on the membranes of wake-active neurons which promote arousal causing wakefulness and alertness. When adenosine binds to A1 receptors on the membranes of wake-active neurons it causes them to polarize thus inhibit wakefulness. A2A receptors are located on the membranes of sleep-active neurons. The combined effect of A1 and A2A activity mediates the sleep-inducing effects of adenosine [38]. As an adenosine antagonist, caffeine binds to the A1 and A2A adenosine receptors in the central nervous system which prevent their function on sleep promotion. This interaction with the adenosine receptors is responsible for caffeine’s role in increased alertness. In the central nervous system, A2A receptors are co-located with the D2 dopamine subtype receptors. Medium spiny neurons (MSNs) are the principal neurons in the striatum. These neurons express both adenosine and dopamine receptors, and they are a key component of the basal ganglia and play a crucial role in regulating timing processes [39, 40]. MSNs receive inputs from various cortical regions, including the prefrontal cortex (PFC), motor cortex, and sensory cortices. These cortical inputs provide information about the current state of the environment, including sensory cues and motor plans, which are essential for timing processes [40]. Research shows that the administration of dopaminergic drugs causes changes in the timing of functions [41, 42]. Psychostimulants that increase the level of dopamine in the synapse are shown to speed time perception, therefore causing overestimations of duration [1, 43–45]. On the other hand, drugs that cause a decrease in the dopamine levels in the synapse cause underestimation of time. Therefore, it slows temporal perception [41, 42, 46, 47].

Under certain conditions, adenosine and dopamine receptors can form dimers, where two receptor molecules come together and bind to each other [22]. Dimerization of adenosine and dopamine receptors can alter receptor signaling and downstream cellular responses. For example, dimerization may change the affinity of receptors for their respective ligands (adenosine or dopamine), leading to altered receptor activation and signaling pathways [9, 48, 49]. The dimerization of adenosine and dopamine receptors can have various functional consequences, including desensitization to the corresponding neurotransmitter, which can lead to changes in the overall responsiveness of cells to adenosine and dopamine signaling. Dimerization can also facilitate cross-talk between different signaling pathways. This cross-talk can influence cellular responses to adenosine and dopamine and interactions with other neurotransmitter systems [50–52].

Our results show caffeine speeds up temporal perception when administered in 9.6–19.2 mg/kg doses. Speeding up of temporal perception is presumably due to an increase in the dopamine levels in the synapse [1, 43–45]. We observed no effect on temporal perception when caffeine is administered in 39.4–96.0 mg/kg. Research shows the effects of L-DOPA (a precursor to dopamine) and dopamine receptor agonists are increased by non-selective adenosine receptor antagonists, such as caffeine and theophylline [22, 23]. These interactions mimic reduced dopamine levels, therefore slowing down the temporal perception. We speculate that as an adenosine receptor antagonist, caffeine (between doses 9.6–19.2 mg/kg) speeds up temporal perception by causing heterodimerization of adenosine and dopamine receptors. However, this effect diminishes as the dose increases (38.4–96.0 mg/kg).

We suggest that this dose-dependent effect of caffeine on temporal perception is like the Yerkes-Dodson Law, at the cellular level (53). In general, the Yerkes-Dodson Law states that task performance increases from low to moderate levels of physiological arousal, but that performance deteriorates at higher levels. The dose-dependent effects of caffeine have been observed in other studies. For example, Kaplan et al. observed that a low/moderate (250mg) dose of caffeine led to overall positive subjective outcomes (i.e., elation, peacefulness, pleasantness) while a higher dose (500mg) led to more negative outcomes (i.e., tension, anxiety, etc.) [52]. In addition, the 250mg dose led to increased performances on a tapping speed test and a digit symbol substitution test while the 500mg dose led to either small or no improvements over a placebo condition at various time points after administration of the caffeine. Several other studies have found parallel non-linear relationships between dose of caffeine and a variety of behavioral measures [52–54]. Therefore, we speculate an inverted-U shaped dose-response, where either too little or too much caffeine has no effect on temporal perception but moderate doses (9.6–19.2 mg/kg) impairs timing through speeding up temporal perception. Since this interaction is not well understood yet, we recommend further experimentation to investigate the effects of adenosine subtype receptors on time perception.

Data Addendum includes three tabs. The first is a description of the data and the experimental design of the two experiments. The second tab contains the data for Experiment 1 while the third tab contains the data for Experiment 2.

## Supporting information

S1 Data (XLSX)
